# Supplementary material for: Traveling and Pinned Fronts in Bistable Reaction-Diffusion Systems on Networks
Source: PLoS One. 2012 Sep 28;7(9):e45029. doi: 10.1371/journal.pone.0045029 (PMC3461022; doi:10.1371/journal.pone.0045029)
Supplement: Supporting Information S1 — The results of the numerical simulations of the bistable Schlögl model (3) for scale-free networks are provided. Traveling fronts and stationary localized patterns are reported for networks with mean degree and sizes or nodes. The observed stationary pattern is compared with the mean-field bifurcation diagram. (PDF) [file pone.0045029.s001.pdf]

Supporting Information S1

In this supporting information, results of numerical simulations of the bistable Schlögl model (3) for scale-free networks with mean degree $\langle k \rangle = 6$ and sizes $N = 150$ or $N = 500$ are reported. The model (3) with the parameters $r_1 = 1$ and $r_3 = 3$ is chosen; the parameter r_2 and the diffusive constant D were varied in the simulations.

Both traveling and pinned fronts were observed. To initiate a traveling front, a node at the periphery with the degree $k = 3$ was set into the active state r_3, whereas the rest of the nodes were in the passive state r_1. This initial configuration generated a front which spread over the entire network.

Front propagation is seen in Fig. S1, where the nodes are grouped according to their distance from the first activated node and the average value ρ_h of the activator density u in each group is plotted as a function of the distance h. Three snapshots of the traveling front at different times are displayed. At $t = 0$, the activation is localized on one node. By time $t = 10$, it spreads to the second neighbors of the original node. At $t = 21$, the activation extends to the fifth neighbors of the original node, covering almost the entire network. Note that a definite traveling front is observed only while the activation is still close to the origin. At the final stage, the front rapidly broadens and the transition to the final uniform active state is quickly taking place.

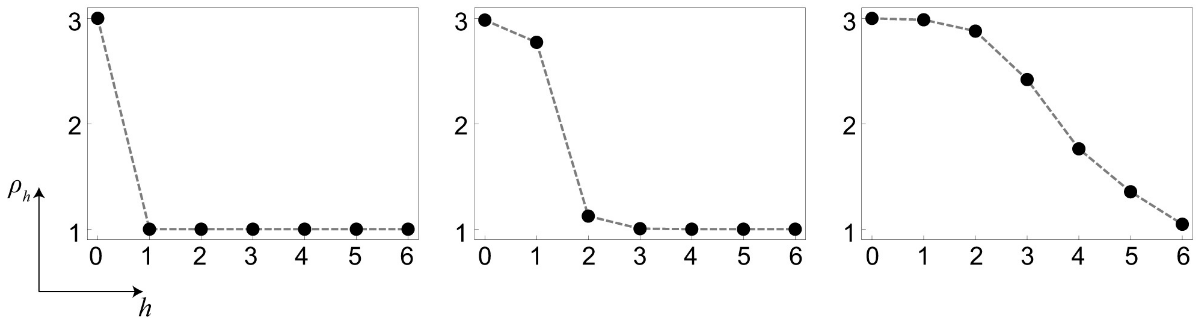

Figure S1. Activation front in a scale-free network with mean degree $\langle k \rangle = 6$ and size $N = 500$. Three consequent snapshots of activity patterns at times $t = 0, 10, 21$ are displayed. Quantity ρ_h is the average value of the activator density u in the subset of network nodes located at distance h from the node which was initially activated. The model parameters are $r_1 = 1, r_2 = 1.2, r_2 = 3$ and the diffusion constant is $D = 0.1$.

When one of the hub nodes was initially activated, a spreading activation front could not be produced. In this case, retracting fronts were observed, if a compact group of nodes with large degrees was initially activated. For weak diffusive coupling, stationary localized patterns were also found, either by appropriate choosing initial conditions, or when traveling fronts was getting pinned at some nodes so that the spreading activation could not reach all network nodes. Two examples of such stationary patterns are shown in Fig. S2.

The mean-field approximation could be used to describe self-organized stationary patterns on the scale-free networks. The mean-field computed in the numerical simulations for the stationary pattern shown in Fig. S2A was equal to $Q = 1.68$. Substituting this value into the Eq. (16) we obtain the bifurcation diagram of a single node coupled to this mean-field. The stationary pattern is compared with the mean-field bifurcation diagram in Fig. S3B. The curves are predictions of the mean-field approximation and the crosses show the simulation data. We see that the data points are distributed along the two stable branches of the bifurcation diagram, indicating good agreement with the mean-field approximation.

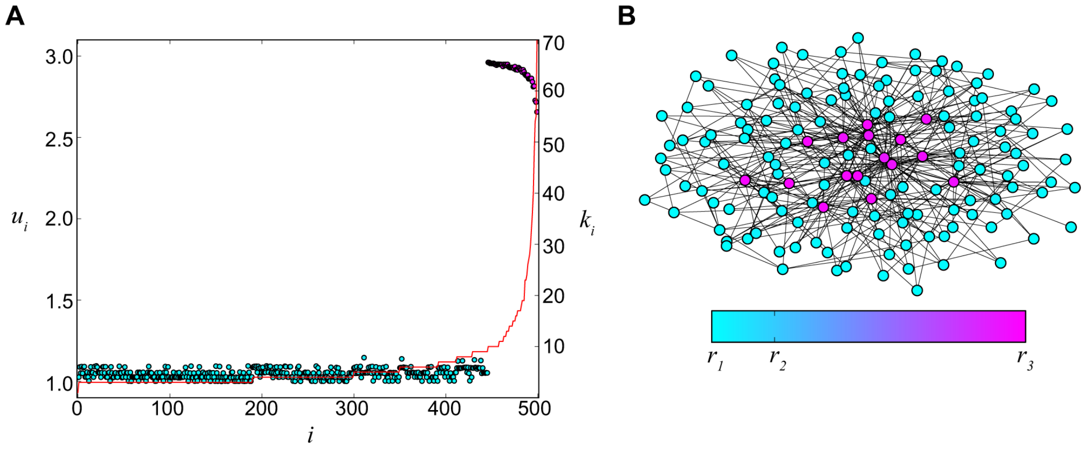

Figure S2. (A) Dependence of the activation level u_i on the degrees k_i of the nodes i for a stationary pattern in the scale-free network of size $N = 500$ and mean degree $\langle k \rangle = 6$. The red curve shows the degrees of the nodes. (B) Stationary pattern in the scale-free network of size $N = 150$ and mean degree $\langle k \rangle = 6$. The nodes with higher degrees are located closer to the center. The nodes are colored according to their activation level, as indicated in the bar. The parameters are $r_1 = 1, r_2 = 1.4$ and $r_3 = 3$; the diffusion constant is $D = 0.01$.

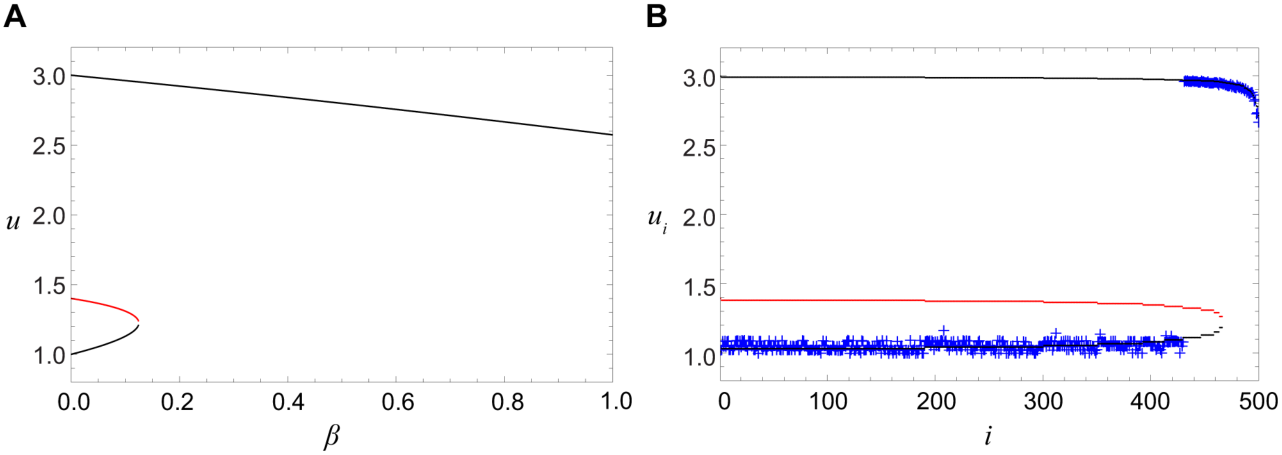

Figure S3. (A) The bifurcation diagram of Eq. (16) for the mean field $Q = 1.68$. (B) Activity distribution in the stationary pattern in the scale-free network of size $N = 500$ and mean degree $\langle k \rangle = 6$ at $D = 0.01$ is compared with the activator levels u predicted by the mean-field theory for the mean field $Q = 1.68$ of the numerically computed pattern. Blue crosses show the simulation data. Black and red curves indicate stable and unstable fixed points of the mean-field equation (16). The other parameters are $r_1 = 1, r_2 = 1.4, r_3 = 3$.
